# The Study on the Effects of *Pimpinella anisum* on Relief and Recurrence of Menopausal Hot Flashes

**Published:** 2012

**Authors:** Fatemeh Nahidi, Nourossadat Kariman, Masoumeh Simbar, Faraz Mojab

**Affiliations:** a *Faculty Member of Midwifery Department Shahid Beheshti University, Tehran, Iran*; b *Pharmaceutical Sciences Research Center, School of Pharmacy, Shahid Beheshti University of Medical Sciences, Tehran, Iran.*

**Keywords:** Menopause, Menopausal women, Vasomotor hot flash, Postmenopausal hot flash, Herbal medicine, *Pimpinella anisum*

## Abstract

With respect to the high incidence of hot flash in postmenopausal women and the controversies regarding its treatment, this double-blind clinical trial was conducted to determine the effects of *Pimpinella anisum *on hot flashes in these women referring to rural and urban health centers of Qazvin Province in 2009.

Seventy-two women with hot flashes were randomly selected according to the predetermined criteria and divided into two experimental and control groups. Their medical records at health centers were used for sampling. Each woman in the experimental group took a capsule containing 330 mg *Pimpinella anisum *3 times a day while in the control group, women took 3 capsules, each containing 330 mg potato starch, over 4 weeks and after that, they were following up for 4 weeks. Before taking the capsules, they were assessed for 2 weeks about the frequency and severity of hot flashes. Data were collected through a questionnaire and an information form. Content validity method was used for validity of the tools. ANOVA and Student›s t-test were applied for statistical analysis. In the experimental group, the frequency and severity of hot flashes before the treatment were 4.21% and 56.21% and, after that, were 1.06% and 14.44% at the end of the fourth week respectively. No change was found in the frequency and severity of hot flashes in the control group. The frequency and severity of hot flashes was decreased during 4 weeks of follow up period. *P. anisum *is effective on the frequency and severity of hot flashes in postmenopausal women.

## Introduction

Hot flash is one of the most common and problematic complications of menopause (1). It is a subjective feeling of heat lasting about 3 min in upper part of the body (2). It is preceded by palpitation and feeling of pressure in the head and may be associated with flushing, weakness, syncope or vertigo. It usually ends with perspiration and cold feeling. Almost 50% of women experience hot flash within 3 months after menopause. It occurs mostly at nights and may awaken the sufferer. The resultant poor quality of sleep may lead to chronic fatigue manifested by irritability, poor concentration and impaired memory (1).

Hot flash and perspiration are not lifethreatening but can cause distress and discomfort. They also affect on household affairs and leisure time (3). Since hot flashes result from abrupt drop of estrogen in menopause, the treatment of choice is estrogen replacement. According to the latest studies, replacement therapy can result in stroke (4), endometrial cancer, cholelithiasis, hypertension, breast cancer, thrombophlebitis, and glucose intolerance (1). With respect to different physical and emotional complications of current interventions such as estrogen therapy, choosing a safe and efficient method is essential. Thus, medicinal herbs can gain high acceptance. *P. anisum *is a herb with estrogenic properties (6, 7) and it seems that herbal compounds containing estrogen can decrease hot flashes. This double-blind clinical trial was conducted to determine the effects of *P.* *anisum *on hot flashes of postmenopausal women referring to rural and urban health centers of Qazvin province in 2009.

## Experimental

Seventy-two subjects were first selected by purposive sampling method through reviewing their family records at Mohammadieh urban health center and rural centers in Hosseinabad, Basher, and Bekandi Villages. First of all, they were contacted to come to the centers. In case they didn’t show up, one of the researchers went to their homes to know if they desire to participate in the study. Then, those who wanted to participate were randomly divided into two equal experimental and control groups, each of which contained 36 postmenopausal women. Inclusion criteria are: 1) being between 45-60 years old, 2) having experience of hot flashes, 3) being literate or having a literate family member, 4) having history of amenorrhea for at least 1 year and at most 3 years. They were excluded from the study in case of undergoing hormone therapy; taking anticoagulant, antidepressive or anxiolytic drugs; experiencing stressful events such as divorce, death of close family members and sensitivity to *P. anisum*. Questionnaires 1 and 2 (filled in two parts by the researchers) as well as 2 information forms were used for data collection. Questionnaire 1 included demographic information, gynecologic history and questions regarding exercise, severity of hot flashes and coping strategies. Information forms 1 and 2 were completed by the subjects over 2 weeks before the intervention to assess dietary habits, frequency and severity of hot flashes. In this study, used the *Pimpinella* *anisum*, seeds (aniseeds/Fam. Apiaceae) it is the kind of anise. The fresh and dried aniseeds were purchased from herbal market in Tehran in June 2008 and after being identified at Medicinal Plant Lab., School of Pharmacy, by TLC and comparison with standard, we became certain of the seeds (Ref. Iranian Herbal Pharmacopoeia Committee, *Iranian Herbal Pharmacopoeia*, Iranian Health Ministry, Tehran 2002, 81.).The seeds were powdered and extracted by ethanol 96%. Solvents were evaporated in vacuum and the *P. anisum *extract was mixed by potato starch and filled in capsules, each of which contains 330 mg of *Pimpinella anisum*. Placebo capsules had the same shape and color. They were filled by 330 mg potato starch. Each woman in the experimental group took a capsule of *Pimpinella anisum *3 times a day. In contrast, in the control group, women took 3 placebo capsules. Both groups took the capsules during 4 weeks and recorded the frequency and severity of hot flashes in information forms. After the treatment, the number of hot flashes and its severity were recorded in both groups during the 4-week follow up period. During these 10 weeks, the information form 1 was weekly given to the subjects and then collected from them for documenting their daily foods in order to assess drug interaction with materials containing phytoestrogens. Additionally, the information form 2 was given to the subjects and collected from them for documenting the frequency and severity of hot flashes every week. Drug sensitivity was assessed by giving a 330 mg capsule of *Pimpinella anisum *to the subjects before the intervention and monitoring for any side effects. Thirty capsules were provided to each woman every week to ensure the consumption of drugs. At the end of each week, remained capsules were collected from the subjects and questionnaire 2 was completed by the researcher. To control the intervening factors, the two groups were matched in terms of age, duration of amenorrhea, frequency and severity of hot flashes. A scale and a metal meter were used for measuring weight and height. Content and test-retest methods were applied for validity and reliability (r = 0.94) of the tools respectively. Reliability of the scale was identified after 10 times of comparison with a control weight.

Different statistical tests including ANOVA with repeated measurements and Student’s t-test were used for data analysis.

## Results

Both groups showed no significant differences in terms of age, body mass index (BMI), age of menarche, interval of the last menstruation, gravidity, number of live births, women’s education and their spouses’ education, marital status, occupation of spouse, family income, residential status, history of abortions and stillbirth, drinking cold liquids, exercise and walking, conflicting with husband, interval of hot flash onset, time of hot flashes, ability for daily activities and relieving interventions (Table 1). 

**Table 1:** Distribution of the subjects in terms of demographic and gynecologic characteristics, and their coping strategies with hot flashes in the two groups

** Groups**		***Pimpinella anisum *** **(n = 36)**	**Placebo (n = 36)**
**Variables**			
**Age (years)** **BMI** **Age of menarche (years)** **Interval from the last menses (year)** **Gravidities** **No. of live births**		51.77 ± 3.08 27.26 ± 3.09 13.66 ± 0.98 2.45 ± 0.84 7.19 ± 4.75 5.52 ± 3.16	51.50 ± 2.80 26.99 ± 3.02 13.33 ± 0.95 2.33 ± 0.77 5.91 ± 3.31 5.02 ± 2.51
**Educational level**	**Illiterate** **Primary** **Secondary** **High school or higher**	13 (36.1%) 14 (38.9%) 6 (16.7%) 3 (8.3%)	18 (50%) 11 (30.6%) 4 (11.1%) 3 (8.3%)
**Spouse’s educational level**	**Illiterate** **Primary** **Secondary** **High school or higher**	12 (34.3%) 11 (31.4%) 7 (20%) 5 (14.3%)	11 (32.4%) 15 (44.1%) 4 (11.8%) 4 (11.7%)
**Marital status**	**married** **widow**	35 (97.2%) 1 (2.8%)	34 (94.4%) 2 (5.6%)
**Spouse’s occupation**	**Businessman** **Employee** **Worker** **Jobless**	6 (17.1%) 6 (17.1%) 13 (37.2%) 10 (28.6%)	19 (55.9%) 3 (8.8%) 11 (32.4%) 1 (2.9%)
**Family income ($/month)**	**≤ 100-200** **201-350** **> 351**	14 (38.9%) 20 (55.6%) 2 (5.5%)	14 (38.9%) 20 (55.6%) 2 (5.5%)
**Residential status**	**Private** **Rental** **Relatives**	32 (88.9%) 3 (8.3%) 1 (2.8%)	36 (100%) - -
**History of abortion**	**Yes** **No**	14 (38.9%) 22 (61.1%)	12 (33.3%) 24 (66.8%)
**Stillbirths**	**Yes** **No**	13 (37.1%) 22 (62.9%)	9 (25%) 27 (75%)
**Drinking cool liquids**	**Yes** **No** **Occasionally**	14 (38.8%) 2 (5.6%) 20 (55.6%)	12 (33.3%) 2 (5.6%) 22 (61.1%)
**Conflict with spouse**	**Never** **Occasionally**	19 (52.8%) 17 (47.2%)	20 (55.5% 16 (44.5%)
**Exercise**	**Yes** **No**	21 (58.3%) 15 (41.7%)	21 9 (58.3%) 15 (41.7%)
**Time of walking (min.)**	**≤ 15-30** **31-60**	11 (42.8%) 12 (57.2%)	6 (28.5%) 15 (71.5%)
**Interval from hot flash onset ( years)**	**≤ 1.5-2** **2.1-2.5** **2.6-3**	18 (50%) 3 (8.3%) 15 (41.7%)	20 (55.6%) 3 (8.3%) 13 (36.1%)
**Time of hot flashes**	**Day** **Night**	9 (25%) 27 (75%)	7 (19.4%) 29 (80.6%)
**Ability for daily activity**	**Yes** **No**	26 (72.2%) 10 (27.8%)	26 (72.2%) 10 (27.8%)
**Interventions to Relieve or decrease**	**Yes** **No**	19 (52.8%) 17 (47.2%)	15 (41.7%) 21 (58.3%)

There was no significant difference in the frequency of hot flashes at the beginning of the study between the groups. The frequency in the experimental group reached from 4.21 before the intervention to 3.60 in the 1st week and to 1.06 in the 4th week. The differences of means were statistically significant (p < 0.001) before the intervention and in the 1st, 2nd, 3rd and 4th week after it. The corresponding figures in the placebo group were 4.24 and 4.28 before and after the treatment respectively with no significant differences (Table 2).

**Table 2:** Means of hot flash frequency before and after (1st, 2nd, 3rd, and 4th week) treatment and (8th week) follow up in the two groups

**Time of treatment**	**Pimpinella anisum**		**Placebo**		**Test result**
	mean	SD	mean	SD	
**Before**	4.21	1.84	4.24	1.87	NS
**1st week**	3.60	1.70	4.27	1.71	p < 0.001
**2nd week**	2.50	1.04	4.20	1.52	p < 0.001
**3rd week**	1.63	0.80	4.27	1.55	p < 0.001
**4th week**	1.10	0.61	4.38	1.73	p < 0.001
**8th week**	1.60	1.76	4.22	1.75	p < 0.001
**Test result over time**	p < 0.001		NS		

**Figure 1:**
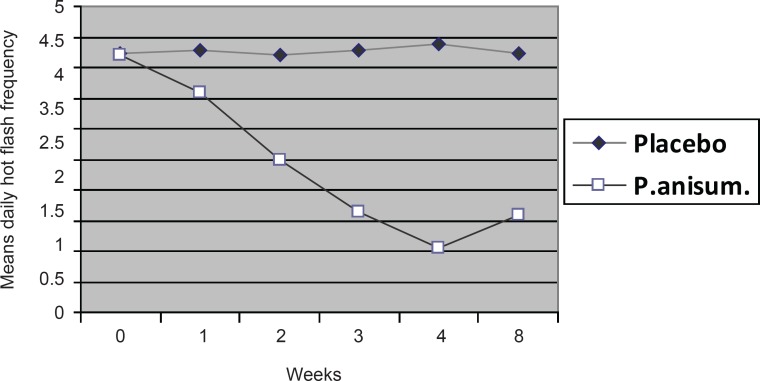
Means of daily hot flash frequency before and after (1st, 2nd, 3rd, 4th and 8th week) the treatment in the two groups

The significant differences between the frequency of hot flashes over the weeks of intervention and follow up in the *Pimpinella* *anisum *and placebo groups are also shown in Figure 1 (p < 001).

There was no significant difference between the means of hot flash severity in the groups according to t-test. The means of hot flash severity in the *Pimpinella anisum *group were 56.21, 49.32, and 14.44 before the intervention, in the 1st and 4th week respectively. ANOVA with repeated measurements showed a significant difference between the means of hot flash severity before and after the treatment (p < 0.001), (*i.e.*, after 4 weeks of intervention, hot flash severity was decreased significantly). However, the difference of severity before and after the treatment in the control group was not significant (Table 3).

**Table 3:** Mean percentage of hot flash severity before and after (1st, 2nd, 3rd, and 4th week) the treatment and (8th week) follow up in the two groups

**Time of treatment**	**Pimpinella anisum**		**Placebo**		**Test result**
	mean	SD	mean	SD	
**Before**	56.21	14.89	53.33	14.9	NS
**1st week**	49.32	14.76	56.09	15.75	p < 0.001
**2nd week**	33.01	12.82	55.55	15.55	p < 0.001
**3rd week**	22.56	11.96	54.25	15.73	p < 0.001
**4th week**	14.44	10.63	56.25	16.41	p < 0.001
**8th week**	20.21	12.62	53.55	16.55	p < 0.001
**Test result over time**	p < 0.001		NS		

**Figure 2:**
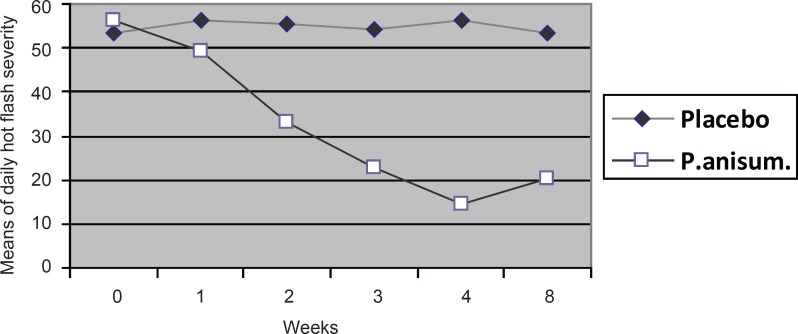
Means of daily hot flash severity before and after (1st, 2nd, 3rd, 4th and 8th week) the treatment in the two groups

Figure 2 shows a significant difference between hot flash severities in the groups over the weeks of intervention and follow-up, (*i.e.* *Pimpinella anisum *was significantly reduced the severity during 4 weeks and its effects maintained for 4 weeks of follow-up). 

Findings revealed that 25% and 30%.5 of the subjects in *Pimpinella anisum *and placebo groups respectively suffered from severe hot flashes before the intervention while, after the treatment, the corresponding figures were 0% and 25%. About 11.1% and 13.9% of the women before the intervention as well as 61.1% and 5.6% of them after it experienced mild hot flashes in the experimental and control groups respectively (Table 4).

**Table 4:** Distribution of hot flash severity before and after (1st, 2nd, 3rd, and 4th week) the treatment in the two groups.

**Groups**		**Before the treatment**		**After the treatment**	
**Hot flash severity**		**No.**	SD	**No.**	**%**
**Anise**	**Mild**	4	11.1	22	61.1
	**Moderate**	23	63.9	14	38.9
	**Severe**	9	25	0	0
	**Total**	36	100	36	100
**Placebo**	**Mild**	5	13.9	2	5.6
	**Moderate**	20	55.6	25	69.4
	**Severe**	11	30.5	9	25
	**Total**	36	100	36	100

## Discussion and Conclusion

In general, the results showed that *Pimpinella anisum *had desirable effects on severity and frequency of hot flashes. Jeffery *et al. *reported the effects of phytoestrogens in clover juice on reducing hot flash frequency (8). Evelyn *et al. *indicated that soy abstract can decrease the hot flash frequency (9). Hayrick *et al. *also reported the effects of hop extract on reducing the severity and frequency of hot flashes as the present study (10). Yari *et al. *observed the influence of soya on alleviating hot flash severity in their study (11). Abbaspour *et al. *found great effects of soya on severity and frequency of daily hot flashes (12). Vernhoven *et al. *reported that the phytoestrogens in either of two medicinal drugs, isoflavone and* Cimicifuga*, reduced hot flash severity inside the groups while no reduction was observed between the groups in terms of alleviating menopausal symptoms in climacteric stage (13). On the other hand, Cheri *et al. *found no alleviating effect of soy phytoestrogens on postmenopausal hot flashes in women with breast cancer (14, 15). This may be due to the stress as an aggravating factor for hot flashes as a result of cancer and the resultant emotional imbalance leading to anxiety and depression. Most of the subjects in the study had had moderate hot flashes before the treatment while they experienced mild symptoms after the intervention with no case of severe hot flashes. In the study of Yari *et al.*, most subjects had suffered from severe hot flashes before the intervention while they experienced moderate hot flashes after it and a group still experiencing severe symptoms (11). In the present study, the frequency and severity of daily hot flashes decreased after 1 week of treatment while in Abbaspour *et al.*, the decrease started from the third week (12). In women taking estrogen for hot flashes, the decrease can be experienced 2 to 4 weeks after the treatment (1). With respect to the rapid expectation of clients to observe the positive effects of drugs, *Pimpinella anisum *may have more preference than the other herbal or chemical drugs due to its more rapid effects. One of the strengths of this study is having 4-week follow-up period after the treatment with *Pimpinella anisum *and placebo to determine maintenance *Pimpinella anisum *effects on menopausal hot flashes. The results showed that *Pimpinella anisum *can control the symptoms long after the consumption. In this study, all foods taken by the subjects were documented and evaluated to prevent interactions of *Pimpinella anisum *with compounds containing phytoestrogens. Therefore, due to this control, this study can argue that *Pimpinella anisum *is a medicinal herb effective on reducing the severity and frequency of hot flashes. Its administration is completely easy, noninvasive, safe and efficient based on this study. Therefore, this drug may be recommended in different forms for relieving the postmenopausal problems of women. In addition, it can be suggested that women should be educated and informed about all benefits and disadvantages of different therapies for hot flashes before choosing a therapy. It can be useful to inform women about the effects of stress on aggravating hot flashes and the effects of the intake of liquids and cool environment to soothe them, which were clearly evident in the present study. Consequently, using herbal or chemical drugs should be then accompanied with instruction regarding managing the stress, changing lifestyle and having foods with phytoestrogens. It can be concluded that these, altogether, can be effective on resolving their

emotional, social and familial problems due to hot flashes. Blood estrogen levels before, during and after the study were not measured. In addition, the severity of hot flash was assessed only by women’s expressions. Since a few studies have been conducted concerning the effects of *Pimpinella anisum* on hot flashes with controversies regarding the consumption and influences of phytoestrogens in this regard, further research with longer duration and measuring blood estrogen levels can better indicate the effects of phytoestrogen in *Pimpinella anisum *on hot flashes.
